# Whipple’s disease mimicking rheumatoid arthritis can cause misdiagnosis and treatment failure

**DOI:** 10.1186/s13023-017-0630-4

**Published:** 2017-05-25

**Authors:** Cornelia Glaser, Siegbert Rieg, Thorsten Wiech, Christine Scholz, Dominique Endres, Oliver Stich, Peter Hasselblatt, Walter Geißdörfer, Christian Bogdan, Annerose Serr, Georg Häcker, Reinhard E. Voll, Jens Thiel, Nils Venhoff

**Affiliations:** 1 Department of Rheumatology and Clinical Immunology, Medical Center. Faculty of Medicine, University of Freiburg, Hugstetter Str. 55, 79106 Freiburg, Germany; 2 Department of Medicine II, Gastroenterology, Hepatology, Endocrinology, and Infectious Diseases, Faculty of Medicine, University Hospital Freiburg, Hugstetter Str. 55, 79106 Freiburg, Germany; 3 Institute of Pathology, Nephropathology Section, University Hospital Hamburg, Eppendorf, Martinistraße 52, 20246 Hamburg, Germany; 4 Private Practice for Rheumatology, Bertoldstraße 8, 79098 Freiburg, Germany; 5 Section of Experimental Neuropsychiatry, Department of Psychiatry, Faculty of Medicine, University of Freiburg, Hauptstraße 5, 79104 Freiburg, Germany; 6 Department of Neurology, Medical Center, University of Freiburg, Breisacher Straße 86, 79110 Freiburg, Germany; 7 Mikrobiologisches Institut - Klinische Mikrobiologie, Immunologie und Hygiene, Universitätsklinikum Erlangen, Friedrich-Alexander-Universität (FAU) Erlangen-Nürnberg, Wasserturmstraße 3-5, 91054 Erlangen, Germany; 8 Institute for Microbiology and Hygiene, Faculty of Medicine, University of Freiburg, Hermann-Herder-Str. 11, 79104 Freiburg, Germany

**Keywords:** *Tropheryma whipplei*, Whipple’s disease, Infectious arthritis, Rheumatoid arthritis, Treatment resistant arthritis, Erosive arthritis

## Abstract

**Background:**

Whipple’s disease, a rare chronic infectious disorder caused by *Tropheryma whipplei*, may present with predominant joint manifestations mimicking rheumatoid arthritis (RA).

**Methods:**

A retrospective single-center cohort study of seven patients was performed. Clinical symptoms were assessed by review of medical charts and Whipple’s disease was diagnosed by periodic-acid-Schiff-stain and/or *Tropheryma whipplei*-specific polymerase-chain-reaction.

**Results:**

Median age at disease onset was 54 years, six patients were male. Median time to diagnosis was 5 years. All patients presented with polyarthritis with a predominantly symmetric pattern. Three had erosive arthritis. Affected joints were: wrists (5/7), metacarpophalangeal joints (MCPs) (5/7), knees (5/7), proximal interphalangeal joints (PIPs) (3/7), hips (2/7), elbow (2/7), shoulder (2/7). All patients had increased C-reactive-protein concentrations, while rheumatoid factor and anti-CCP-antibodies were absent, and were initially (mis)classified as RA-patients according to EULAR/ACR-criteria (median DAS28 4.3). Six patients received antirheumatic treatment consisting of prednisone with methotrexate and/or leflunomide, three were additionally treated with at least one biologic agent (abatacept, adalimumab, etanercept, rituximab, tocilizumab). Most patients showed insufficient treatment response. In all patients *Tropheryma whipplei* was detected in synovial fluid by polymerase-chain-reaction; in three patients the diagnosis of Whipple’s disease was further ascertained by periodic-acid-Schiff-staining. Gastrointestinal symptoms and other extra-articular manifestations were absent, mild or non-specific. Treatment was initiated with trimethoprin/sulfamethoxazole in five and doxycycline/hydroxychloroquine in two patients and had to be adapted in five patients. Finally, all patients had good treatment responses with improvement of arthritis and extra-articular manifestations.

**Conclusion:**

Whipple’s disease is rare and can mimic rheumatoid arthritis. Especially patients with seronegative rheumatoid arthritis with a prolonged disease course and insufficient treatment response should be reevaluated for Whipple’s disease.

## Background

Whipple’s disease (WD) is a very rare disorder caused by a chronic infection with *Tropheryma whipplei (TW),* a bacterium found ubiquitously in the environment [1, 2]. Joint involvement is a common feature of WD and found in 40–80% of the patients during the prodromal stage of the disease [3, 4]. Patients frequently develop chronic, rheumatoid factor- and anti-CCP antibody-negative, mostly non-erosive arthritis that predominantly affects the large joints. The clinical presentation as inflammatory arthritis often leads to a misdiagnosis of spondyloarthritis, rheumatoid arthritis, or gouty arthritis [3–5]. Without antibiotic treatment the natural course of WD may be fatal; erroneously induced immunosuppressive treatment may even accelerate the clinical progression of the disease [6]. Later stages of the disease are in most cases characterized by systemic symptoms like fever, weight loss and diarrhea. Neuropsychiatric and cardiac involvement has been described in about one-quarter of WD-patients [7]. Furthermore, skin manifestations, mesenteric lymph node and lung involvement have been described [4, 8]. Diagnosis of WD can be established by periodic acid-Schiff (PAS) staining of inclusion bodies within lamina propria macrophages in biopsies of the small intestine and by polymerase chain reaction (PCR). The high specificity and sensitivity of *TW*-specific PCRs or of 16S and 23S *TW-*rRNA followed by sequence analysis allows testing of various tissues including sterile body fluids such as cerebrospinal fluid or synovial fluid [9, 10]. The aim of our study was to describe the clinical phenotype, diagnostic work-up, treatment and outcome of WD patients presenting with polyarticular arthritis classified by rheumatologists as rheumatoid arthritis (RA) according to the 2010 ACR/EULAR criteria [11]. Our data indicate that especially polyarticular manifestations of WD mimicking RA can lead to misdiagnosis and potentially false treatment decisions.

## Methods

In this retrospective single-center cohort study patients with diagnosis of WD after previously suspected seronegative rheumatoid arthritis were included. All patients were recruited at the Department of Rheumatology and Clinical immunology of the University Medical Center Freiburg, Germany from 2010 to 2015. The study was approved by the ethics committee of the Albert-Ludwigs-University, Freiburg (file No. 191/11, 46/04). Written informed consent according to the declaration of Helsinki was obtained from all patients. Definitive diagnosis of WD was established by PAS staining on tissue samples and/or specific PCR assays (23S rDNA- und 16S-23S ribosomal intergenic spacer PCR) for *TW* of body fluids or tissue samples.

For all patients detailed information including age, sex, date of first clinical symptoms and of final diagnosis, prior medical history and previous immunosuppressive treatment were collected from medical charts. Clinical presentation was determined in detail with respect to number, size and pattern of the involved peripheral and axial joints. Joint destruction was evaluated by radiography, MRI and ultrasound of the affected joints. Generalized symptoms like fever, weight loss, night sweat, fatigue, and lymphadenopathy were recorded. All patients were screened for gastrointestinal disease manifestations and for cardiac or neurological involvement.

Laboratory studies included blood cell counts, hemoglobin, C-reactive protein (CRP), erythrocyte sedimentation rate (ESR), ferritin, and albumin. Immunological laboratory testing included rheumatoid factor, anti-CCP-antibodies, ANA, and ANCA. Furthermore, serum immunoglobulin concentrations for IgG, IgA, and IgM were evaluated. When available, results of synovial fluid analysis (SFA), synovia biopsy and cerebrospinal fluid (CSF) analysis (cell count, total protein, oligoclonal bands) were recorded. All patients received endoscopic work-up by esophagogastroduodenoscopy including biopsies of the small intestine.

## Results

Within five consecutive years we diagnosed Whipple’s disease in seven patients, all characterized by polyarticular arthritis which had led to the misdiagnosis of RA. All but one patient were male. Median age at diagnosis was 54 years (range 44–68). The median time between the onset of clinical symptoms and diagnosis of WD was 5 years (range 1–180 months). Initially all patients were diagnosed having seronegative RA and all but one patient received immunosuppressive treatment. All patients suffered from arthritis or at least arthralgia. At the time point of WD diagnosis six patients presented with polyarthritis with a chronic intermittent disease course.

Retrospectively, in 6 of the 7 patients first disease manifestation was an intermittent, asymmetrical arthritis with oligoarthritis in 5 of the 7 patients. At first presentation in our center five patients had polyarthritis affecting the hands; in others the classical symmetric pattern was suggestive for RA. Most frequently affected joints were the following: wrists (5/7), metacarpophalangeal joints (MCP) (5/7), knees (5/7), proximal interphalangeal joints (PIPs) (3/7), hips (2/7), elbow (2/7), shoulder (2/7). In one patient (#6) WD was diagnosed after only 4 weeks of arthralgia. In all patients arthritis was initially classified as RA using the 2010 EULAR/ACR classification criteria [11]. In three patients (#1, 2, 7) diagnosis was further corroborated by the finding of radiologic erosive joint destruction, described by board certified radiologists in at least one joint (Table 1). Retrospectively, radiographs have been reevaluated by a specialist in WD and interpreted as a typical pattern of articular involvement of Whipple’s disease [12] with severe carpal destructive changes with fusion and ankylosis at both wrists and minor involvement of MCP and PIP. The finding of bone erosions did not correlate with disease duration, but was found more frequently in patients with an inflammatory disease course indicated by high serum CRP concentrations. A median disease activity score (DAS28) of 4.3 (range 2.8–6.3) seemed to be indicative of active disease in all patients. All patients were negative for rheumatoid factor and anti-CCP-antibodies. Furthermore ANA and ANCA screening was negative in all patients. All but one patient (#6) had received immunosuppressive therapy consisting of prednisone, combined with synthetic DMARDs, most frequently methotrexate (*n* = 5) or leflunomide (*n* = 4). Three patients (#1, 2, 7) were treated with at least one biologic agent (adalimumab, etanercept, abatacept, rituximab, or tocilizumab). Median treatment duration with sDMARDs were 15 months (IQR 5–50 months) and 12 months (IQR 7–24 months) with bDMARDs. All patients showed either insufficient or absent treatment responses. In one patient (#1) disease course even deteriorated during therapy with biological DMARDs. Finally, because of inadequate treatment response and persistent joint swelling arthrocentesis was performed in six patients and analysis yielded a positive *TW* PCR in all. As a consequence of the positive *TW* PCR in the synovial fluid, we completed the diagnostic work-up in all patients as described (Table 2). In patient #1 *TW* was detected by PAS-staining and PCR in a synovial biopsy specimen of the knee. Leucocyte count in synovial fluid was within the range of 1000 to 7000 cells/μl reflecting a mild to moderate inflammation of the joints in four patients.

**Table 1:** Epidemiological, clinical and laboratory parameters of WD patients mimicking RA

patient		WD diagnosis		Musculoskeletal manifestation			Laboratory findings			Extra-articular manifestations											
										GI-tract				neurology				cor	skin		
#	sex	age at first clinical manifestation (years)	time to diagnosis of WD (months) Follow-up after diagnosis (months)	Articular manifestation/number of involved joints/pattern of joint involvement	Radiographic findings	ACR/EULAR Criteria DAS28 (ESR)	CRP (mg/L) ESR (mm)	hemoglobin (♀ 11.6–15.5 g/dl) (♂ 13.5–17.6 g/dl)	SFA (cells/μl)	abdominal pain	weight loss	loss of appetite	nausea	depression	dysgeusia	polyneuropathy	headache	pericarditis	hyperpigmentation	erythema	Immuno-suppressive treatment (treatment duration in months)
1	m	38	180 60	Symmetric polyarthritis (12 large, 40 small): wrists, MCP, PIP, atlanto-axial joint, hips, knees	Erosive arthritis	7 6.28	84.5 22/45	7.3	500			X		X	X	X	X			X	Pred (132), SSZ (4), MTX (43), LEF (26), ETN (3), ADA (4), ABA (8), RTX (6), TCZ (3)
2	m	64	47 48	Symmetric polyarthritis (10 large, 2 small): wrists, elbows, hips, knees, ankles	Erosive arthritis	7 3.58	8.2 26/50	12.8	1100					X							Pred (35), MTX (5), LEF (1), ADA (4), TCZ (3)
3	m	45	56 36	Asymmetric polyarthritis (1 large, 5 small): MCP joints and left knee	Non erosive arthritis	5 4.46	4.9 27/57	11.9	7200									X			-
4	m	34	124 56	Asymmetric polyarthritis (3 large, 3 small): MCP, PIP, left wrist, right knee	Non erosive arthritis	5 4.34	18.3 24/54	14.7	7800							X					Pred (6), MTX (4)
5	m	57	129 57	Symmetric arthritis (3 large, 2 small): shoulders, wrists, and left hip	Osteo-proliferation on PIP and MTP, no erosions	5 4.1	37.2 53/89	12.5	No data							X					Pred (4), MTX (8), LEF (7)
6	m	55	1 30	Symmetric arthralgias (2 large, 3 small): MCP, PIP, elbows, vertebral column, sterno-costal joints, axial manifestation	Osteopenia of the hands, no erosions	4 2.5	12 22/45	15.1	No data												Pred (2)
7	f	50	42 24	Asymmetric poly-arthritis (6 large, 4 small): shoulders, wrists, MCPs, knees, and MTPs	Erosive arthritis	5 4.5	106 44/78	10.9	1300	X	X	X	X						X		Pred (33), MTX (27), LEF (8), ABA (12)

*Abbreviations*: *ABA* abatacept, *ADA* adalimumab, *CRP* C-reactive proteine, *DAS28* disease activity score 28, *ESR* erythrocyte sedimentation rate, *ETN* etanercept, *GI-tract* gastrointestinal tract, *Hb* hemoglobine, *LEF* leflunomide, *MCP* metacarpophalangeal joints, *MTP* metatarsophalangeal joints, *MTX* methotrexate, *pred* prednisone, *PIP* proximal interphalangeal joints, *WD* whipple’s disease, *RTX* rituximab, *SFA* synovial fluid analysis, *SSZ* sulfasalacine, *TCZ* tocilizumab

**Table 2:** Diagnostic evaluation for Whipple’s disease

Pat #	PAS staining		PCR			
	Small intestine	Synovialis	Small intestine	Synovial fluid	Skin	CSF
1	+	+	+	+	+	n.d.
2	−	n.d.	+	+	n.d.	−
3	−	n.d.	−	+	n.d.	−
4	−	n.d.	−	+	n.d.	−
5	−	n.d.	+	+	n.d.	−
6	+	n.d.	+	n.d.	n.d.	n.d.
7	+	n.d.	+	+	n.d.	+

*Abbreviations*: +, positive; −, negative, *CSF* cerebrospinal fluid, *PAS* periodic acid-Schiff staining, *PCR* polymerase chain reaction, *n.d* not done

Extra-articular manifestations of WD were frequent (Table 1) but mainly mild and not indicative for WD. In particular gastrointestinal symptoms occurred only infrequently and were rather mild. None of the patients showed “classic” symptoms with dyspepsia and diarrhea and only one patient reported on previously unexplained weight loss. Endoscopic workup was performed in all patients. Gastrointestinal symptoms were mild or absent even in five patients, in which TW could be detected in small intestinal biopsies. TW was detected in the intestines of only three patients (#2, 6 and 7) by both PAS-staining and PCR, but only one patient (#7) had gastrointestinal symptoms and weight loss. One patient experienced a transient cutaneous rash on his legs. Skin biopsy showed a non-specific lymphocytic tissue infiltration with positive detection of *TW* by PCR. Neuropsychiatric symptoms were mild and non-specific consisting of polyneuropathy, depression, dysgeusia, and headache. In five patients lumbar puncture was performed to exclude cerebral involvement. In none of the patients with putatively WD-associated neurological symptoms *TW* was detectable in the CSF. On the contrary, the only patient with a positive *TW*-PCR in the CSF did not appear to have any neurological symptoms. With respect to laboratory findings all patients developed at least transient and in some cases clinically relevant inflammation with accelerated ESR and CRP concentrations (Table 1). Five patients developed transient (*n* = 3) or chronic (*n* = 2) anemia. None of the patients developed hypoalbuminemia during their disease course.

**Fig. 1:**
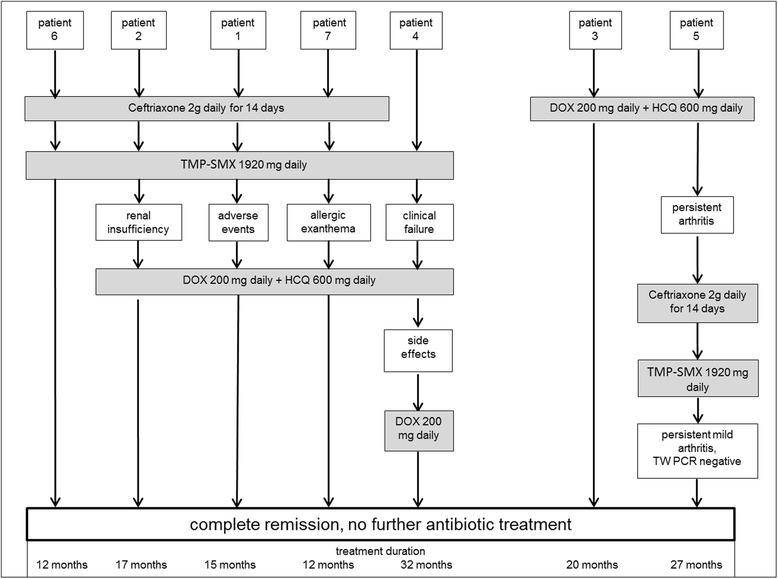
Antibiotic treatment and disease course. Legend: Depicted are antibiotic treatment and disease course, including treatment duration, response to treatment, adverse events, and change of treatment in the seven patients with WD. Abbreviations: DOX, doxycycline; HCQ, hydroxychloroquine; TMP-SMX, trimethoprim-sulfamethoxazole

Antibiotic treatment was initiated in all patients following the diagnosis of WD. Treatment regimens and disease course are depicted in Fig. 1. Four patients were initially treated with ceftriaxone 2 g/day for 14 days. Five patients received long-term treatment with trimethoprim/sulfamethoxazole (800/160 mg twice daily). Two patients received treatment with doxycycline 200 mg/day and hydroxychloroquine (200 mg three times daily). In five patients (#1, 2, 4, 6, 7) the antibiotic regimen had to be modified due to side effects or insufficient treatment response: One of them (#4) received trimethoprim/sulfamethoxazole twice daily without initial application of ceftriaxone because there were no signs of neurological involvement. He relapsed with arthritis and recurrent detection of *TW* in the synovial fluid by PCR, so treatment with trimethoprim/sulfamethoxazole was supposed to have failed and was changed to doxycycline combined with hydroxychloroquine. After 7 months hydroxychloroquine therapy was discontinued because of mild dizziness. Doxycycline was continued for another 9 months and then discontinued in a state of complete remission. Another patient (#5) treated with doxycycline plus hydroxychloroquine had to discontinue treatment after 15 months because of recurrent arthritis. Treatment was switched to ceftriaxone 2 g/d for 14 days followed by trimethoprim/sulfamethoxazole twice daily for 1 year and resulted in a good and long-lasting treatment response for at least 18 months after the end of treatment. PCR for *TW from* synovial tissue during follow-up after antibiotic treatment remained negative. Thus, all patients exhibited a good treatment response with striking improvement of arthritis and all extra-articular manifestations.

## Discussion

Diagnosis of Whipple’s disease is often delayed, especially in cases without any clinical evidence of gastrointestinal involvement. WD predominantly affects middle-aged white men, which was also evident in our cohort. All patients in our cohort suffered from joint manifestations mimicking seronegative RA. While most patients suffered from an asymmetrical, intermittent arthritis at disease onset, they later presented with symmetric arthritis in more than half of the cases, when they were first admitted to our department. Apart from the pattern of involved joints the suspected diagnosis of RA was further supported by the chronic disease course, involvement of the small joints in all, and joint erosions in three patients. As the clinical manifestations were considered to be compatible with a diagnosis of RA, in all but one patient anti-rheumatic treatment was initiated. Joint involvement is a very common feature in WD and occurs in 40–80% of patients at least transiently [2–4]. In contrast to the patients of this case series, joint manifestations in WD have been most often described as arthralgia or arthritis, predominantly affecting the large joints like knees, wrists, ankles, hips and shoulders, whereas involvement of the small joints have been by far less often reported [13]. In our patients also small joints were affected and erosive joint manifestations were observed in almost half of these patients (compared to a frequency below 10% described in other cohorts [4, 13]). Of note, we could detect erosions only in small joints. The fact that bone erosions of the small joints are rather characteristic for RA and are part of the 1987 ACR-classification criteria for RA might have led to the wrong diagnosis in the reported patients [14]. As mentioned above it is possible that destructive arthritis is misinterpreted even by radiologists and that a wrong interpretation of radiographs may lead to wrong diagnoses. Thus, both rheumatologists and radiologists should increase their awareness for rare diseases like WD as a reason for erosive bone changes.

Another distinctive feature that might have contributed to a delay in diagnosing WD was the absence of gastrointestinal symptoms in almost all of our patients. In most cohorts of WD patients reported to date, 80–90% of the patients develop gastrointestinal symptoms with weight loss, diarrhea or both, at least at later stages of the disease. In some patients the phenomenon of delayed gastrointestinal symptoms for up to 6 years after the onset of WD-associated arthritis was described [15], suggesting that some of our patients might have developed gastrointestinal symptoms if remained untreated. Others described the occurrence of gastrointestinal manifestations under immunosuppressive treatment in undiagnosed WD-patients [6]. In particular, treatment with TNF-alpha-inhibitors was reported in several cases to unmask WD by development of gastrointestinal symptoms [6, 16]. The patients in our cohort did not develop any relevant gastrointestinal manifestations even on long-term immunosuppressive therapy. Besides prednisolone or synthetic DMARDs, biologic DMARDs including TNF-alpha-inhibitors were used. Interestingly, Patient #1 had the longest disease course and immunosuppressive therapy and exhibited the most severe phenotype. But even in this case worsening of the arthritis and general symptoms developed slowly over time and were thus misinterpreted as the natural disease course of treatment-resistant RA. In retrospective, the lack of a sufficient treatment response with respect to arthritis should have prompted differential diagnoses, but unfortunately mainly led to switching of the immunosuppressive treatment regimen or even to treatment escalation to at least one or even more biological agents. Treatment-resistant rheumatoid arthritis, especially if seronegative, should lead to careful reevaluation. Arthritis that worsens during immunosuppression requires rigid exclusion of infection. Joint infections with bacteria of low pathogenicity such as atypical mycobacteria also need to be considered in patients with clearly established diagnosis of RA. Aspiration of synovial fluid or synovial biopsy for detection of bacteria by culture and PCR are required. Unfortunately, in case of treatment failures the availability of a growing number of new anti-rheumatic drugs often leads to a switch of medication rather than to a reevaluation of the diagnosis.

WD is a very rare condition and diagnostic procedures are at least partially invasive. Recently, an algorithm for the diagnosis of Whipple’s arthritis including PCR of synovial fluid, stools and saliva has been proposed [17]. The patients of the present study, however, presented with inflammatory arthritis and therefore arthrocentesis was performed. As PCR was positive for *TW* in all tested patients of our cohort, the diagnostic work-up in all patients was completed but no further *TW*-diagnostic on saliva and stools was performed. Our data indicate, that performing *TW*-PCR during diagnostic work-up of synovial fluid as performed in our patients can be crucial for the diagnosis of TW-arthritis. In classic WD, PAS-positive foamy macrophages can be found in the lamina propria and the specificity of the result can be increased by detection of *TW* by PCR [9, 10]. Despite no or only mild gastrointestinal symptoms in three of our patients, PAS-positive macrophages were visible in duodenal biopsies and positive PCR-testing confirmed these findings. Especially in patient #1, who was characterized by a long disease course with a large number of insufficient immunosuppressive regimens, *TW* was detectable by PCR at all investigated sites, even in the skin. Detection of *TW* in the skin of patients with classic WD even without apparent skin manifestations has been described and may also be helpful for diagnosis when WD is suspected [8]. The three patients of our study with PAS-positive macrophages in duodenal biopsies and positive PCR-testing for *TW* can be classified as definite classic WD [15]. Duodenal biopsies of the remaining four patients were negative in PAS-staining and only two patients had a positive duodenal *TW*-PCR. The fact that in all patients PCR-testing was positive in synovial fluid from at least one inflamed joint supports the diagnosis of localized *TW* infection in these individuals [15]. As mentioned, PCR-testing of saliva and stool samples to screen for *TW* in patients with unexplained articular pain, weight loss and unclear abdominal lymphadenopathy has been proposed by Fenollar et al. [15]. In patients with mere *TW*-arthritis without any gastrointestinal symptoms *TW*-PCR of saliva or stool specimens may be difficult to interpret given the possibility of asymptomatic carriers.

Finally, in all patients WD was treated with either ceftriaxone followed by long-term trimethoprim/sulfamethoxazole or doxycycline combined with hydroxychloroquine. Treatment duration ranged between 12 and 20 months following the current treatment recommendations [2].

In one patient doxycycline was discontinued because of recurrent arthritis. Changing the antibiotic treatment regimen resulted in a prompt clinical improvement. In this patient an immune reconstitution inflammatory syndrome (IRIS) – as previously described in whipple’s disease- may be an explanation for the reoccurrence of arthritis [18]. As this patient refused to undergo another joint puncture, a recurrence of the *TW* infection cannot be ruled out. Two patients had to be treated for 20 or 32 months, respectively, because treatment had to be changed several times due to side effects or insufficient clinical response. Ultimately, all patients achieved complete remission and required no further treatment during follow-up. All patients are still under close monitoring with regular visits at least twice per year in our outpatient clinic. The excellent response to antibiotic therapy with no relapse after treatment cessation strongly supports the diagnosis of WD-arthritis, even in the two patients that were positive for *TW* only in the PCR analysis of synovial fluids.

## Conclusions

In conclusion, WD is a rare but important differential diagnosis of rheumatoid arthritis as it may exclusively manifest as an inflammatory polyarthritis without gastrointestinal symptoms. In these cases, *TW*-PCR-testing of synovial fluid is critical to establish the diagnosis. Hence, seronegative RA - especially in male patients - with a prolonged disease course and insufficient treatment response should be reevaluated for WD. In these patients PCR-analysis of synovial fluid may be the most sensitive diagnostic procedure to detect *TW*; histopathological evaluation of PAS-stained duodenal biopsies alone is not sufficient to exclude WD.

## Abbreviations

CRP: C-reactive protein
CSF: Cerebrospinal fluid
ESR: Erythrocyte sedimentation rate
MCPs: Metacarpophalangeal joints
PAS: Periodic acid-Schiff staining
PCR: Polymerase chain reaction
PIPs: Proximal interphalangeal joints
RA: Rheumatoid arthritis
SFA: Synovial fluid analysis
TW: Tropheryma whipplei
WD: Whipple’s disease
